# Impairment of Several Immune Functions and Redox State in Blood Cells of Alzheimer’s Disease Patients. Relevant Role of Neutrophils in Oxidative Stress

**DOI:** 10.3389/fimmu.2017.01974

**Published:** 2018-01-11

**Authors:** Carmen Vida, Irene Martinez de Toda, Antonio Garrido, Eva Carro, José Antonio Molina, Mónica De la Fuente

**Affiliations:** ^1^Facultad de Biología, Universidad Complutense de Madrid, Madrid, Spain; ^2^Instituto de Investigación Hospital Universitario12 de Octubre (i+12), Madrid, Spain; ^3^Centro de Investigación Biomédica en Red sobre Enfermedades Neurodegenerativas (CIBERNED), Madrid, Spain

**Keywords:** Alzheimer’s disease, immunosenescence, immune function, oxidative stress, inflammation, blood cells, neutrophils, mononuclear cells

## Abstract

Since aging is considered the most risk factor for sporadic Alzheimer’s Disease (AD), the age-related impairment of the immune system (immunosenescence), based on a chronic oxidative-inflammatory stress situation, could play a key role in the development and progression of AD. Although AD is accompanied by systemic disturbance, reflecting the damage in the brain, the changes in immune response and redox-state in different types of blood cells in AD patients have been scarcely studied. The aim was to analyze the variations in several immune functions and oxidative-inflammatory stress and damage parameters in both isolated peripheral neutrophils and mononuclear blood cells, as well as in whole blood cells, from patients diagnosed with mild (mAD) and severe AD, and of age-matched controls (elderly healthy subjects) as well as of adult controls. The cognitive decline of all subjects was determined by Mini-Mental State Examination (MMSE) test (mAD stage was established at 20 ≤ MMSE ≤ 23 score; AD stage at <18 MMSE; elderly subjects >27 MMSE). The results showed an impairment of the immune functions of human peripheral blood neutrophils and mononuclear cells of mAD and AD patients in relation to healthy elderly subjects, who showed the typical immunosenescence in comparison with the adult individuals. However, several alterations were only observed in severe AD patients (lower chemotaxis, lipopolysaccharide lymphoproliferation, and interleukin (IL)-10 release; higher basal proliferation, tumor necrosis factor (TNF)-α release, and IL-10/TNF-α ratio), others only in mAD subjects (higher adherence), meanwhile others appeared in both mAD and AD patients (lower phytohemaglutinin lymphoproliferation and higher IL-6 release). This impairment of immune functions could be mediated by: (1) the higher oxidative stress and damage also observed in blood cells from mAD and AD patients and in isolated neutrophils [lower glutathione (GSH) levels, high oxidized glutathione (GSSG)/GSH ratio, and GSSG and malondialdehyde contents], and (2) the higher release of basal pro-inflammatory cytokines (IL-6 and TNF-α) found in AD patients. Because the immune system parameters studied are markers of health and rate of aging, our results supported an accelerated immunosenescence in AD patients. We suggest the assessment of oxidative stress and function parameters in peripheral blood cells as well as in isolated neutrophils and mononuclear cells, respectively, as possible markers of AD progression.

## Introduction

Alzheimer’s disease (AD) is the most common neurodegenerative disorder and one of the major causes of senile dementia in later life. Epidemiological studies have revealed that AD is a multifactorial disease, with a complex interplay of environmental and genetics factors, which helps explain its variable clinical presentation (1, 2). Traditionally, AD has been classified into hereditary and sporadic forms. The hereditary form is linked with several genes and typically presents an earlier age of onset. By contrast, the sporadic form, which is the most common cause of AD (>95% of cases), has a later age of onset and a stronger association with aging, the major risk-incurring variable (1, 2). Due to the increase in mean life span, this pathology is exponentially increasing and is estimated that the prevalence of AD may reach >115 million worldwide by 2050 (3). Despite the enormous social, economic and health care impact, public health care systems do not have the medical therapies necessary to address AD. Moreover, the diagnosis of individual with early AD is not easy, and it is usually diagnosed in late stages (even postmortem), by which time the available treatments are not effective (4). For this reason, it is crucial to identify biomarkers of early diagnosis of AD that can help in the development of effective therapeutics and prevention methods.

Neuropathologically, AD is a progressive and irreversible brain disorder characterized by the extracellular accumulation of amyloid-β (Aβ) plaques and intracellular neurofibrillary tangles (NFTs) of hyperphosphorylated tau protein, associated with neuronal cell death and synaptotoxicity (5). Additional changes such as brain atrophy, mitochondrial dysfunction, increase in oxidative stress, and neuroinflammation can also occur in the brains of people with AD (6, 7). However, the mechanism of AD pathogenesis and progression still remain unclear. During the last few years, a higher number of studies have demonstrated that AD is also accompanied by a systemic disturbance, reflecting the damage in the brain (8–10). Thus, several studies support the involvement of an immune-related systemic alteration in AD, which includes changes in both innate and adaptive immune systems (11–15). Given the bidirectional communication between the immune and the nervous systems, and due to how the mediators of peripheral immune cells can influence the central nervous system (CNS) (16), it is not surprising that the age-related immunological variations can modify this neuroimmune communication network, contributing to the progressive cognitive impairment in AD (16). Indeed, numerous studies have shown changes in the distribution and reactivity of immune cells in the blood of AD patients (12, 17–21). This seems to cause a chronic inflammation in the periphery, which can be propagated through CNS immune cells, leading to neuroinflammation, which contributes to the cognitive deficits associated with AD (16, 22, 23).

Given that aging is accompanied by a decline of the nervous and the immune systems, as well as of their communication (24), which contributes to the deterioration of homeostasis and health, AD can be understood in the context of aging (25). Since advanced age is considered the most consistent risk factor for sporadic AD (1), the age-related dysregulation of the immune system, which is denominated immunosenescence, should be considered to understand the development of this pathology (11, 16, 17, 25–27). Immunosenescence involves restructuring changes in both innate and adaptive immune functions, which negatively affect the health of older adults, increasing susceptibility to infections and mortality (24, 28). Thus, there is an age-related decrease in several leukocytes functions, such as phagocytosis, chemotaxis, or stimulated proliferation, as well as an increase in other functions, such as adherence capacity to tissues and spontaneous lymphoproliferations, among others (24, 28–30). In AD patients, several immune functions are hampered in relation to healthy individuals with the same chronological age (8, 11, 19, 31–34), suggesting that this immune deterioration may be considered as a pathogenically relevant factor in this disease (33). Thus, it is assumed the advanced immunosenescence in AD patients, with remarkable immunological changes compared to healthy elderly subjects, which could contribute to AD pathology (11, 16, 17, 26, 33). Among these changes in AD patients the most relevant have been observed in the adaptive immune system. In fact, as also occurs in immunosenescence, several authors have reported changes in T and B lymphocyte differentiation and subpopulation distribution in peripheral blood of AD subjects, as well as an altered proliferative T lymphocytes response (11, 33, 34). Nevertheless, the results are inconsistent since increased or decreased lymphoproliferation and percentages in T and B lymphocytes, as well as no alterations in these immune parameters have been detected in AD patients (11, 33, 34). Regarding innate immunity, abnormalities in the function of natural killer (NK) cells (31, 32), a decreased phagocytosis of Aβ in peripheral macrophages (19), and an altered release of pro-inflammatory cytokines [e.g. interleukin-(IL)-6, tumor necrosis factor (TNF)-α, etc.] (8) have been shown in AD patients in relation to age-matched controls. However, the data of human studies are still scarce, preliminary, and contradictory.

According to the oxidation–inflammation theory of aging, the basis of immunosenescence is a chronic oxidative-inflammatory stress situation (a progressive imbalance between higher endogenous levels of oxidant and inflammatory compounds and lower antioxidant and anti-inflammatory defenses) (24, 28). In fact, immune cells continuously generate oxidants and inflammatory compounds to carry out their defensive functions; however, if the overproduction of oxidant compounds is not well-controlled by the antioxidant defenses, an alteration occurs in the redox balance, leading to an oxidative stress situation, which causes oxidative damage in biomolecules (e.g., lipids, proteins, etc.), inducing remarkable negative consequences on cellular functioning (24, 28). In addition, the chronic inflammation, which is characterized by excessive production and release of pro-inflammatory cytokines and chemokines, also leads to signaling cascades that trigger the production of oxidant compounds and depletion of antioxidants (35, 36). Therefore, since oxidation and inflammation are interlinked processes, an active inflammatory response by immune cells can lead to cellular damage due to oxidant overproduction, which can also recruit other inflammatory cells amplifying the cellular damage (35, 36). In this context, there is much evidence that indicates the strong involvement of inflammation and oxidative stress and damage in the onset, progression, and pathogenesis of AD (37–40). Thus, as occurs in aging, an enhanced oxidative stress has been observed in the brain (39, 40), as well as in peripheral tissues and cells (e.g., blood cells) (27, 41, 42) from AD patients. Indeed, many studies show both overproduction of oxidant compounds and impairment of antioxidant systems, as well as increased of oxidative damage (e.g., lipid peroxidation, proteins and DNA oxidation) in several brain regions of patients with both severe AD and mild cognitive impairment (MCI) (40, 43, 44). These markers of oxidative damage were also detected in high levels in peripheral blood and cerebrospinal fluid (CSF) obtained from both MCI and AD patients, together with decreased plasma levels of non-enzymatic antioxidants and impaired activity of antioxidant enzymes. Moreover, peripheral levels of the pro-inflammatory cytokines (e.g., IL-6 or TNF-α) have been described to be higher, whereas anti-inflammatory cytokine levels (e.g., IL-10 and IL-4) are lower in patients with AD compared to age-matched controls (27, 42, 43, 45–47). Interestingly, the fact that high levels of peripheral markers of oxidative-inflammatory stress were also detected in individuals with MCI, support the hypothesis that oxidative stress and inflammation are early events in the pathogenesis of AD, and precede Aβ deposit and onset of AD symptoms, as has been seen in experimental models of AD (48, 49). However, although several studies have evaluated oxidative stress parameters in both the early and advanced stages of human AD, the findings are controversial, as well as the relation between oxidative stress and cognitive performance, in mild and severe AD subjects, not yet being fully understood.

The use of animal models is particularly useful to study the molecular mechanisms involved in the development of AD and to identify potential therapeutic targets. In the last few decades, several transgenic animal models of AD have been developed to reproduce the neuronal pathology and behavioral symptomatology of human AD. The triple-transgenic mouse model (3xTg-AD) represents a unique animal model that closely mimics neuropathological manifestations, Aβ-plaques, and NFTs, in an age-dependent and region-specific manner like those in the human AD brain (50). Interestingly, as occurs in aging, it has been described that 3xTg-AD mice also suffer a pronounced and accelerated impairment in the neuroimmune network (16, 26), as well as a marked deterioration of several immune functions and an increased oxidative stress in different types of immune cells (e.g., peritoneal leukocytes) in comparison to control mice (16, 26, 51, 52). These alterations were observed in both early and advanced stages of the AD neuropathology, as well as before the onset establishment of AD (16, 26, 51, 52). Given that these changes are characteristics of prematurely and chronologically aged subjects (28, 30, 53), the premature immunosenescence observed in the 3xTg-AD mice at early stages of AD could explain the shorter life span also observed in these animals (52). Interestingly, the immune functions of peritoneal leukocytes of mice have been found to possess similar age-related evolutions to those observed in human circulating immune cells (30). Therefore, since immune functions are good markers of the rate of aging, and their analysis allows the early identification of premature and accelerated aging in humans (28, 30), the assessment of these parameters could be useful peripheral markers of the prodromal and preclinical states of AD (52).

It is also important to note that the most of studies performed in peripheral blood cells, have been carried out using mainly isolated peripheral mononuclear cells (95% lymphocytes and 5% monocytes), which are the most commonly used cells in the clinical setting. However, there are a few studies in which changes in immune function and redox status have been assessed in isolated phagocytes. Since these cells (e.g., neutrophils and macrophages) have been suggested to be the main cells responsible for the chronic oxidative-inflammatory stress associated with immunosenescence (28, 54), they could provide a helpful sample to clarify the molecular mechanisms underlying the impairment of the immune system throughout of AD progression. Moreover, blood, and particularly circulating leukocytes, reflects the major physiological changes in various body organs and systems (47). Therefore, the use of blood cells seems to be more useful in assessing markers that are analyzed in CSF, as well as identifying new markers that could be predictive of AD. Thus, it is possible that the assessment of these immune function parameters in the different types of peripheral blood immune cells, together with the evaluation of their oxidative-inflammatory state, could be useful early peripheral markers of the progression of AD in humans. However, this kind of study in different stages of human AD is still a subject that has scarcely been explored.

With the above in mind, the aim of the present work was to study the changes in several immune functions and inflammatory-oxidative stress and damage parameters in different types of human blood immune cells at early and advance stages of AD progression. To address this study, we performed assays on both isolated peripheral polymorphonuclear (PMN) and mononuclear blood leukocytes, as well as in whole blood (WB) cells, from patients diagnosed with mild and severe AD, and age-matched controls (elderly healthy subjects) as well as adult controls.

## Materials and Methods

### Subjects and Clinical Classification

For this cross-sectional study, a total of 102 volunteers were selected and divided into four experimental groups: adult healthy subjects (*n* = 20), elderly healthy individuals (*n* = 38), mild AD (mAD) patients (*n* = 26), and severe AD patients (*n* = 18). All subjects were recruited by the Neurology Department of the Hospital, 12 Octubre of Madrid, and were tested by a standardized neuropsychological battery. The AD diagnosis was established according to the guidelines of the National Institute on Neurological Disorders and Stroke and the Alzheimer’s Disease and Related Disorders Association (55). Disease severity and normal cognitive function was determined by a clinician’s judgment based on a structured interview with the patient and the results of the Clinical Dementia Rating and the Mini-Mental State Examination (MMSE) tests (56). The mAD stage was established at 20 ≤ MMSE ≤ 23 score and AD stage at <18 MMSE score. Inclusion criteria for cognitively normal elderly healthy subjects were MMSE scores > 27, no history or clinical signs of neurological or psychiatric disease or cognitive symptoms. Demographic details and MMSE test results of the different study groups are summarized in Table 1. All subjects were subjected to a clinical survey and physical examination. Those with a history of cardiovascular disease, cancer, or chronic inflammatory diseases, as well as individuals with current inflammatory alterations (findings of clinical significance in general laboratory parameters) were not included in this study. The consent of the subjects was obtained according to the Declaration of Helsinki, and approval was obtained from the corresponding Research Ethic Committees. Written informed consent was obtained from all participants or representatives.

**Table 1:** Demographic data and neuropsychological test results of adult and elderly healthy subjects, as well as of mild Alzheimer’s disease (mAD) and severe Alzheimer’s disease (AD) patients.

	Adult	Elderly	mAD	AD
*n* (F/M)	20 (10/10)	38 (28/10)	26 (15/11)	18 (13/5)
Age (years)	40.32 ± 8.54	74.34 ± 9.22	76.08 ± 7.46	79.10 ± 6.53
MMSE	n.e.	29.02 ± 0.7	21.02 ± 0.86	16.44 ± 0.84
CDR	n.e.	0	2	3

*F, female; M, male; MMSE, Mini-Mental State Examination Score; CDR, Clinical Dementia Rating; n.e., not evaluated*.

*The data of age and of MMSE score are expressed as mean ± SD, but those of CDR scores are presented as range values*.

### Collection of Peripheral WB Cells and Isolation of Blood Neutrophil and Lymphocytes Cells

Human samples (10 mL) of peripheral blood were collected using vein puncture and sodium citrate-buffered Vacutainer tubes (BD Diagnostic, Spain). Blood extraction was performed between 9:00 a.m. and 10:00 a.m. to avoid circadian variations in immune parameters. On the one hand, 8 mL of peripheral blood was used for isolation of both PMNs (mainly neutrophils) and mononuclear (mainly lymphocytes) leukocytes following a previously described method (57). Thus, neutrophil and lymphocyte cells were isolated using 1.119 and 1.077 g/cm^3^ density Histopaque (Sigma-Aldrich, Spain) separation, respectively. Collected cells were counted (95% of viability determined using trypan blue staining) and adjusted to the corresponding final concentrations for the development of the different assays of redox state and immune functions. The immune functions assays were performed with fresh cells, whereas the redox state assays were assessed in aliquots of frozen cells. These aliquots were stored at −80°C until used. On the other hand, samples of WB cells, which contain the total red blood cells (RBC) together with the total leukocyte populations, were obtained following a previously described procedure (58). For this, 500 µL of the peripheral blood sample was diluted with 500 µL of RPMI 1640 medium without glutamine (Gibco, Burlington, ON, Canada) and 10 µL of gentamicin (0.1 mg/mL in tube). The samples were incubated for 4 h at 37°C in a saturated atmosphere of CO_2_ and humidity. Then, samples were centrifuged at 900 × *g* for 10 min to obtain the WB cell pellets after plasma removal. RPMI 1640 with glutamine (Gibco) was added to the blood cells to make 1 mL, and then several aliquots were prepared for the determination of redox state parameters. These aliquots were stored at −80°C until used.

### Adherent Capacity Assay

For the measurement of adherence capacity of human peripheral blood neutrophils and lymphocytes, we followed a method previously described (59) with some slight modifications. This method mimics, *in vitro*, cellular adherence to endothelium *in vivo*. Briefly, 500 µL of WB diluted 1:1with Hank’s medium was placed in adherence columns consisting of a Pasteur pipet, in which 50 mg of nylon fibers was packed to a height of 1.25 cm. After 10 min, the effluent had drained by gravity, and neutrophils and lymphocytes were counted in this effluent using the Neubauer hemocytometer (microscope, 40×). Aliquots of 100 µL of 50% diluted WB effluent were mixed with 900 µL of Türk’s solution, which has the ability to lyse RBC and differentiate the morphology of PMNs and mononuclear leukocytes. Results were expressed as the number of cells per mm^3^. In addition, another aliquot of 500 µL of WB diluted 1:1 with Hank’s medium was used to count the total number of neutrophils and lymphocytes present in WB, as described earlier. The difference between the number of leukocytes present in the initial mixture and in the effluent, after passing through the adherence column, gives the number of adherent cells. The percentage of adherent neutrophils and lymphocytes, expressed as Adherence Index (AI), was calculated according to the equation:

$$\text{IA}=[\left(\text{leukocytes}/{\text{mm}}^{3}\text{total}-\text{leukocytes}/{\text{mm}}^{3}\text{effluent}\right)/\left(\text{leukocytes}/{\text{mm}}^{3}\text{total}\right]\times 100.$$

### Chemotaxis Assay

The induced mobility or chemotaxis of peripheral blood isolated neutrophils and lymphocytes was carried out following a method previously described (57). Boyden chambers with two compartments separated by a polycarbonate filter (3 µm of diameter; Millipore, Ireland) were used to evaluate the chemotactic index (CI). Aliquots of 300 µL of neutrophil and lymphocyte suspensions (10^6^ cells/mL) in Hank’s medium were deposited in the upper compartment, and aliquots of 400 µL of the chemoattractant agent *N*-formylmethionine-leucyl-phenylalanine (Sigma-Aldrich) at a concentration of 10^−8^ M in the lower compartment of the chambers. The chambers were incubated for 3 h at 37°C, and the filters were fixed and stained with Giemsa’s solution (Sigma-Aldrich). The number of neutrophils and lymphocytes on the lower face of the filter was counted in 20 microscope fields using an immersion objective (×100) and recorded as CI.

### Phagocytosis Assay

Phagocytosis of inert particles (latex beads, 1.1 µm diameter, Sigma-Aldrich) was assayed in phagocytes (isolated blood neutrophils) following a method previously described (57). Neutrophils adjusted to 10^6^ cells/mL were incubated on migration inhibition factor plates (Kartell, Noviglio, Italy) for 30 min at 37°C in a humidified atmosphere. The adherent monolayers obtained were washed with prewarmed PBS solution, and then 200 µL of Hank’s solution and 20 µL of latex beads (1.1 µm diluted to 1% PBS, Sigma-Aldrich) were added. After 30 min of incubation under the same conditions, the plates were washed, fixed with methanol (50%), and stained with Giemsa’s solution (Sigma-Aldrich). The number of particles ingested by 100 neutrophils was counted using an immersion objective (×100) and this was expressed as phagocytic index, while the number of ingesting neutrophils per 100 neutrophils was expressed as phagocytic efficiency.

### NK Cytotoxicity Assay

The NK cell cytotoxicity, which is the main antitumoral protection of the organism, was measured by an enzymatic colorimetric assay (Cytotox 96 TM Promega, Boehringer Ingelheim, Germany) based on the determination of lactate dehydrogenase (LDH) released by the cytolysis of targets cells (human K562 lymphoma cells), using tetrazolium salts (57). Briefly, target cells were seeded in 96-well U-bottom culture plates (Nunclon, Denmark) at 10^4^ cells/well in 1640 RPMI without phenol red (Gibco). Effector cells (mononuclear leukocyte suspensions adjusted to 10^6^ cells/mL) were added at 10^5^ cells/well, obtaining an effector/target rate of 10/1. The plates were centrifuged at 250 × *g* for 5 min to facilitate cell-to-cell contacts and were incubated for 4 h at 37°C. After incubation, LDH activity was measured in 50 μL/well by addition of the enzyme substrate with absorbance recording at 490 nm. The results were expressed as the percentage of tumor cells killed (% lysis).

### Lymphoproliferation Assay

The proliferation capacity of lymphocytes was evaluated by a standard method, previously described (57). The assay was assessed in basal and stimulated conditions using the mitogens phytohemaglutinin (PHA) and lipopolysaccharide (LPS). Aliquots of 200 µL of isolated mononuclear leukocyte suspensions adjusted to 10^6^ cells/mL of complete medium [containing RPMI 1640 enriched with l-glutamine and phenol red and supplemented with 10% heat-inactivated (56°C, 30 min) fetal calf serum (Hyclone, GE Healthcare, USA) and gentamicin (100 mg/mL, Sigma-Aldrich)] were dispensed into 96-well plates (Nunclon, Denmark). 20 µL of complete medium (basal lymphoproliferation), PHA, or LPS (1 µg/mL, Sigma-Aldrich) was added to each well. After 48 h of incubation at 37°C in a sterile and humidified atmosphere of 5% CO_2_, 2.5 μCi ^3^H-thymidine (Hartmann Analytic, Germany) was added to each well. Previously, 100 µL of culture supernatant from each well was collected and stored at −80°C until used for cytokine analysis. After another incubation of 24 h, cells were harvested in a semiautomatic harvester (Skatron Instruments, Norway), and thymidine uptake was measured in a beta counter (LKB, Uppsala, Sweden) for 1 min. The results were calculated as ^3^H-thymidine uptake (counts per minute, cpm) for basal and stimulated (with mitogens) conditions and also were expressed as lymphoproliferation capacity (%) giving 100% to the cpm in basal conditions.

### Glutathione Content Assay

Both reduced [glutathione (GSH)], the main non-enzymatic reducing agent of the organism, and oxidized [oxidized glutathione (GSSG)] forms of glutathione were determined using a fluorometric assay previously described (60). This method is based on the capacity of reaction that GSSG and GSH show with *o*-phthalaldehyde (OPT, Sigma-Aldrich), at pH 12 and pH 8, respectively, resulting in the formation of a fluorescent compound. The assay was evaluated in WB cells (containing total RBC and leukocyte populations), as well as in isolated peripheral blood neutrophils and mononuclear leukocytes. For this, aliquots of frozen WB cells (50 µL) were resuspended in phosphate buffer 0.1 M, pH 7.4 (200 µL) (Sigma-Aldrich), whereas aliquots of isolated neutrophils and lymphocytes adjusted to 10^6^ cells/mL in Hank’s solution were centrifuged at 1,200 × *g* for 10 min at 4°C. All samples were resuspended in phosphate buffer 50 mM containing EDTA 0.1 M, pH 8 (600 µL) (Sigma-Aldrich). Then, samples were sonicated, and after the addition of HClO_4_ (60%, Sigma-Aldrich; 7.5 µL), they were centrifuged at 9,500 × *g* for 10 min at 4°C. Aliquots of supernatants (10 µL) were dispensed into 96-well black plates (Nunc). For GSH measurement, 190 µL of phosphate buffer and 20 µL of OPT (1 mg/mL in methanol) were dispensed in the wells, and the plate was incubated for 15 min in the dark and at room temperature. For the measurement of GSSG, 8 µL of *N*-ethylmaleimide (0.04 M; Sigma-Aldrich) was added to each well to prevent interference of GSH with GSSG. The plate was incubated for 30 min under the same conditions. Then, 186 µL of NaOH (0.1 N) and 20 µL of OPT were incorporated, and the plate was incubated for 15 min in similar conditions. In both GSH and GSSG measurements, the fluorescence emitted by each well was measured at 350 nm excitation and 420 nm emission. Protein content of the samples was determined following the bicinchoninic acid protein assay kit protocol (Sigma-Aldrich), using serum albumin (BSA, Sigma-Aldrich) as standard. Results were expressed as nanomoles of GSH or GSSG per milligram of protein. Moreover, the GSSG/GSH ratio was calculated for each sample.

### Lipid Peroxidation (MDA) Assay

The estimation of MDA, in both blood cells and isolated peripheral blood neutrophils and mononuclear leukocytes, was evaluated using the commercial kit “MDA Assay Kit” (Biovision, Mountain View, CA, USA), which measures the reaction of MDA with thiobarbituric acid (TBA) and the MDA-TBA adduct formation. For this, aliquots of frozen WB cells (100 µL) were resuspended in phosphate buffer 0.05 M, pH 7.4 (200 µL), whereas aliquots of isolated neutrophils and lymphocytes adjusted to 10^6^ cells/mL in Hank’s solution were centrifuged at 1,200 × *g* for 10 min at 4°C. All samples were resuspended in lysis buffer (300 µL) containing butylated hydroxytoluene (0.1 mM, 3 µL), sonicated, and centrifuged again at 13,000 × *g* for 10 min. The supernatants (200 µL) from each sample were added to TBA (600 µL) and incubated at 95°C for 60 min. Samples were cooled in ice for 10 min, and 200 µL of reaction mixture was mixed with 300 µL of *n*-butanol (Sigma-Aldrich) to create an organic phase in which the MDA molecules were to be placed. Samples were centrifuged 10 min at 13,000 × *g* at room temperature, and 200 µL of the supernatants (upper organic phase) was collected and dispensed into a 96-well microplate for spectrophotometric measurement at 532 nm. MDA supplied in the kit was used as standard, and MDA levels were determined by comparing the absorbance of samples with that of the standards. Protein concentration of the samples was measured as described earlier. Results were expressed as nanomoles of MDA per milligram of protein.

### Cytokine Measurement

The IL-6, TNF-α, and IL-10 release was measured in culture supernatants of WB in the absence or presence of LPS following a method previously described (58). Briefly, 500 µL of blood was diluted 1:1 with RPMI 1640 medium without l-glutamine (Gibco) and incubated for 4 h with 10 µL gentamicin (1 mg/mL, Sigma-Aldrich) and 10 µL LPS (250 ng/mL, Sigma-Aldrich) or 10 µL RPMI 1640 medium (basal conditions). Samples were centrifuged, and supernatants were collected and frozen at −20°C until assay. Levels of IL-6, TNF-α, and IL-10 were measured simultaneously by multiplex luminometry (Beadlyte human multiplex cytokine detection system, HCYTOMAG-60K, Millipore, Billerica, MA, USA), with minimum detectable doses of IL-6, TNF-α, and IL-10 under 0.9, 0.7, and 1.1 pg/mL, respectively. The results were expressed as picograms per milliliter.

### Statistical Analysis

Statistical analysis was performed in SPSS IBM, version 21.0 (SPSS, Chicago, USA). All tests were two-tailed, with a significant level of α = 0.05. Data are presented as mean ± standard deviation (SD). Normality of the samples and homogeneity of the variances were checked by the Kolmogorov–Smirnov test and Levene test, respectively. Differences due to age and AD pathology were studied using a one-way analysis of variance followed by *post hoc* tests analysis or the non-parametric Kruskal–Wallis test. The Tukey test was used for *post hoc* comparisons when variances were homogeneous, whereas its counterpart analysis Games-Howell was used with unequal variances when they were not homogeneous. Figures were built using GraphPad Prism 6.

## Results

### Immune Functions in Peripheral Blood Neutrophils and Mononuclear Leukocytes of mAD and AD Patients As Well As Healthy Controls

**Figure 1:**
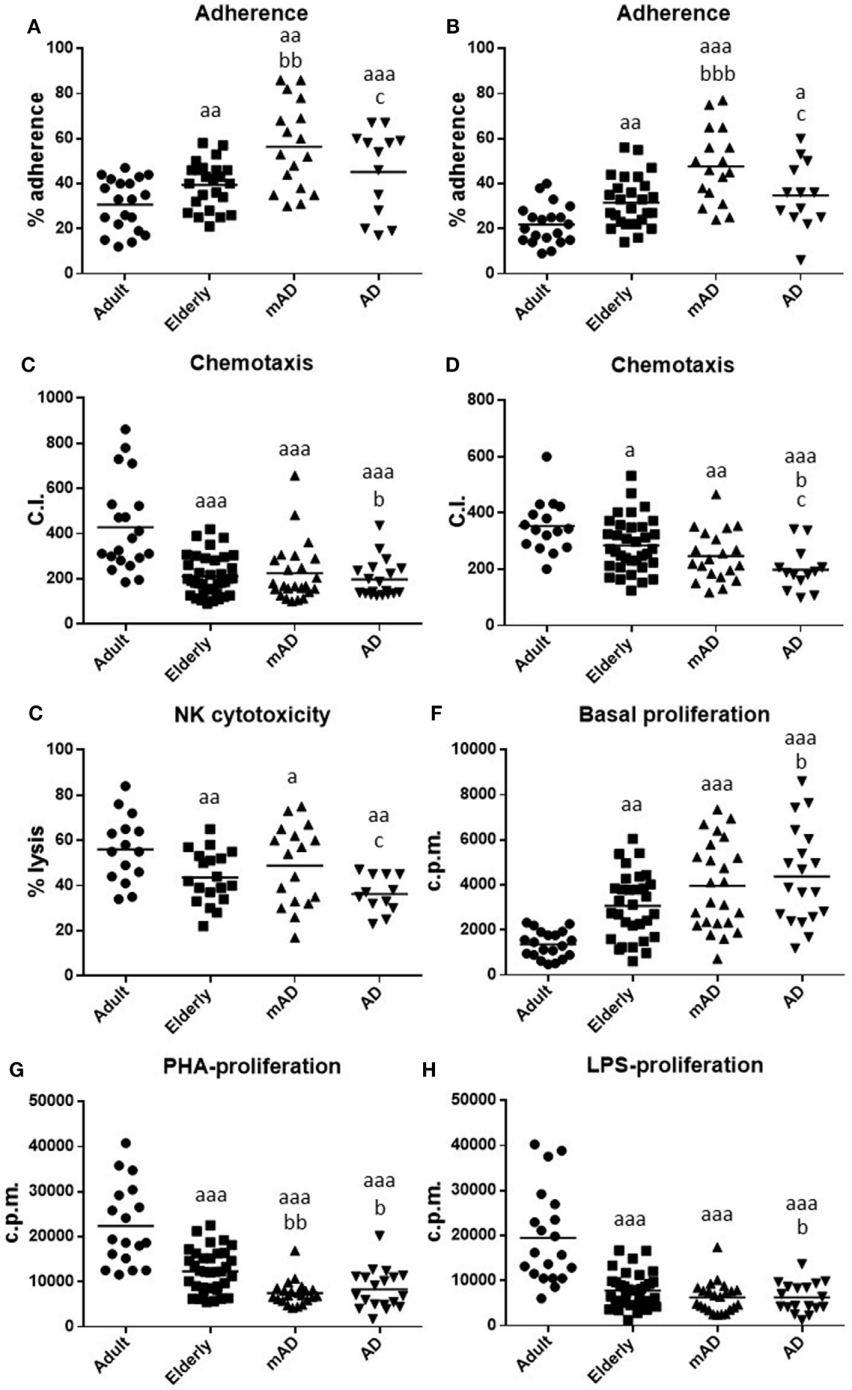
Function parameters in isolated peripheral blood neutrophils and mononuclear cells of mild Alzheimer’s disease (mAD) and Alzheimer’s disease (AD) patients, as well as of adult and elderly healthy subjects. Adherence (%) of neutrophils **(A)** and lymphocytes **(B)**; chemotaxis index (C.I.) of neutrophils **(C)** and lymphocytes **(D)**; natural killer (NK) cytotoxicity (% lysis) **(E)**; proliferation of lymphocytes in basal conditions [48 h incubation; counts per minute (cpm)] **(F)**; proliferation of lymphocytes (48 h incubation; cpm) in response to phytohemagglutinin (PHA, 1 µg/mL) **(G)** and in response to lipopolysaccharide (LPS, 1 µg/mL) **(H)**. Data are shown as the mean (horizontal bar) of 18–38 values corresponding to the number of subjects analyzed in each group (20 adult, 38 elderly, 26 mAD, and 18 AD). Each value is the mean of duplicate assays. a: *P* < 0.05, aa: *P* < 0.01, and aaa: *P* < 0.001 with respect to the value in adult subjects; b: *P* < 0.05, bb: *P* < 0.01, and bbb: *P* < 0.001 with respect to the value in elderly subjects; c: *P* < 0.05 with respect to the value in mAD patients.

The results obtained in the immune functions studied in isolated human blood neutrophils and mononuclear cells obtained from adult and older healthy subjects, as well as from mAD and AD patients are shown in Figure 1 and Table 2. In general, all the immune parameters analyzed in the present work (adherence, chemotaxis, and phagocytosis of neutrophils as well as adherence, chemotaxis and proliferative response of lymphocytes in both basal and stimulated conditions, and the antitumor cytotoxic activity of NK cells) have been shown to deteriorate in aging and AD pathology. However, a different pattern of impaired immune function has been observed between the early and advanced stages of AD.

**Table 2:** Stimulation of proliferation (%) in response to phytohemagglutinin and lipopolysaccharide (1 µg/mL), in isolated peripheral mononuclear cells of adult and elderly healthy subjects, as well as of mild Alzheimer’s disease (mAD) and severe Alzheimer’s disease (AD) patients.

Lymphoproliferation (% stimulation)	Adult	Elderly	mAD	AD
PHA (%)	1,950 ± 232	391 ± 56^a^	198 ± 19^a^^,^^b^	218 ± 36^a^^,^^c^
LPS (%)	346 ± 25	176 ± 13^d^	156 ± 12^c^^,^^e^	167 ± 17^e^

*Data are expressed as mean ± SD of the values corresponding to 20 adult, 38 elderly, 26 mAD, and 18 AD subjects. Each value is the mean of duplicate assays*.

*^a^P < 0.001 with respect to the value in adult subjects*.

*^b^P < 0.01 with respect to the value in elderly subjects*.

*^c^P < 0.05 with respect to the value in elderly subjects*.

*^d^P < 0.05 with respect to the value in adult subjects*.

*^e^P < 0.01 with respect to the value in adult subjects*.

In elderly healthy subjects, in comparison with healthy adults, statistically significant lower values in neutrophil chemotaxis (*P* < 0.001; Figure 1C) and phagocytosis (176 ± 13 and 488 ± 25 P.I., older vs adult, respectively; *P* < 0.001), as well as in the activity of NK cells (*P* < 0.01; Figure 1E), lymphocyte chemotaxis (*P* < 0.05; Figure 1D) and PHA- and LPS-lymphoproliferative response (*P* < 0.001; Figures 1G,H; Table 2) were obtained. In contrast, higher values were also shown in old subjects in neutrophil and lymphocyte adherence *(P* < 0.01; Figures 1A,B), as well as in basal proliferation (*P* < 0.01; Figure 1F).

Regarding AD pathology, there were significant differences in AD patients relative to elderly healthy controls of the same chronological age, as well as in mAD compared to the same controls. Thus, AD patients exhibited higher basal proliferation (*P* < 0.05; Figure 1F) and lower neutrophil and lymphocyte chemotaxis (*P* < 0.05; Figures 1C,D), as well as PHA- and LPS-lymphoproliferative response (*P* < 0.05; Figures 1G,H; Table 2) in comparison to elderly subjects. Interestingly, at the early stage of the disease, mAD patients also showed lower PHA-lymphoproliferation (*P* < *0.01*; Figure 1G; Table 2) than elderly subjects. However, mAD exhibited a significant increase in neutrophil (*P* < 0.01; Figure 1A) and lymphocyte (*P* < *0.001*; Figure 1B) adherence in relation to elderly subjects, which was not observed at the advanced stage of AD. Finally, it should be noted that immune function differences between the early and advanced stages of AD were also observed. Thus, AD patients showed significantly lower neutrophil and lymphocyte adherence (*P* < 0.05; Figures 1A,B), as well as lower chemotaxis of lymphocytes and lower NK cytotoxic activity (*P* < 0.05; Figures 1D,E) than mAD patients.

### Cytokine Production in Response to LPS Stimulation in Blood Leukocytes of mAD and AD Patients As Well As Healthy Controls

Cytokines are major mediators of the complex interactions among immune cells, being responsible for the development and resolve of immune response. Aging is characterized by a chronic low-grade inflammatory state (61), and the maintenance of health relies on the adequate balance of anti-inflammatory and pro-inflammatory compounds (28). For this reason, we analyzed the levels of pro-inflammatory (IL-6 and TNF-α) and anti-inflammatory (IL-10) cytokines secreted *ex vivo* by blood cells incubated for 4 h under LPS-stimulated conditions, in mAD and AD patients, as well as in adult and elderly healthy subjects. The IL-10/TNF-α ratios, which are a good indicator of successful aging and longevity (30), were also calculated.

**Figure 2:**
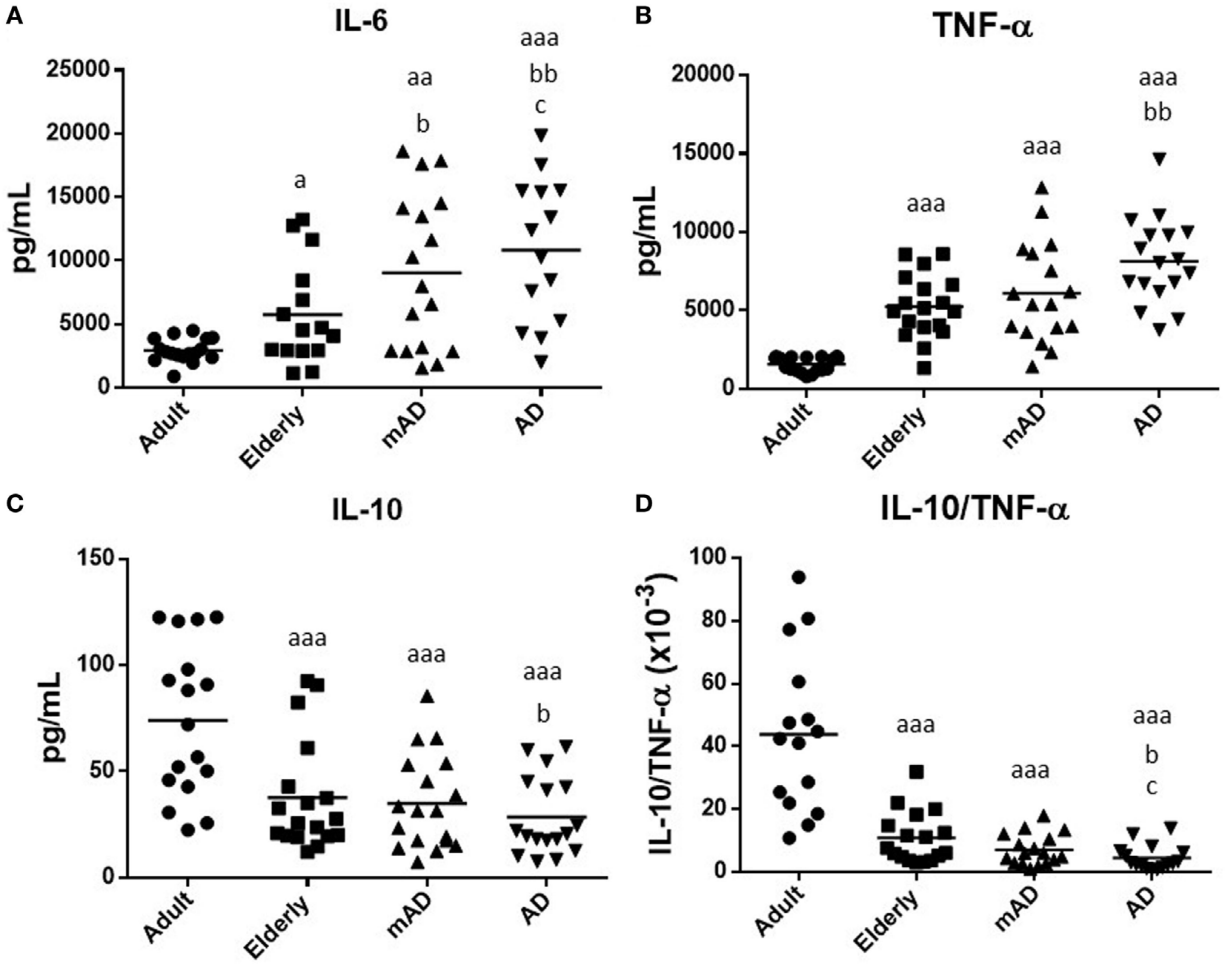
Levels (pg/mL) of cytokines released after 4 h of culture in the presence of lipopolysaccharide (1 µg/mL) by peripheral whole blood cells of mild Alzheimer’s disease (mAD) and Alzheimer’s disease (AD) patients, as well as of adult and elderly healthy subjects. Interleukin (IL)-6 **(A)**, tumor necrosis factor (TNF)-α **(B)**, and IL-10 **(C)**, as well as IL-10/TNF-α ratios **(D)**. Data are shown as the mean (horizontal bar) of the values corresponding to the number of subjects analyzed in each group (20 adult, 18 elderly, 18 mAD, and 18 AD). Each value is the mean of duplicate assays. a: *P* < 0.05, aa: *P* < 0.01, and aaa: *P* < 0.001 with respect to the value in adult subjects; b: *P* < 0.05 and bb: *P* < 0.01 with respect to the value in elderly subjects; and c: *P* < 0.05 and cc: *P* < 0.01 with respect to the value in mAD patients.

In general, the results of our study showed that the release of these cytokines suffers impairments with aging. Moreover, a similar pattern of altered secretion was observed for all investigated cytokines, in AD patients. As shown in Figure 2, under LPS-stimulated conditions, increased levels of IL-6 and TNF-α (*P* < 0.05 and *P* < 0.001, respectively; Figures 2A,B) were observed in the elderly subjects in comparison to adults. This was also accompanied by a marked decrease in IL-10 and the IL-10/TNF-α ratio (*P* < 0.001; Figures 2C,D). A similar cytokine profile was also observed in AD patients in relation to older subjects. Thus, AD patients had higher levels of IL-6 and TNF-α (*P* < 0.01; Figures 2A,B), as well as lower IL-10 release and IL-10/TNF-α ratios (*P* < 0.05; Figures 2C,D) than elderly controls. However, mAD patients only showed a significant increase in the levels of IL-6 (*P* < 0.05; Figure 2A). No differences were observed in TNF-α and IL-10 release and IL-10/TNF-α ratios between mAD and older subjects. Regarding the differences between the different stages of AD, it is important to note that AD patient exhibited a higher IL-6 LPS-induced release (*P* < 0.05; Figure 2A) and a lower IL-10/TNF-α ratio (*P* < 0.05; Figure 4D) than mAD patients.

### Oxidative Stress and Damage Parameters in Isolated Peripheral Blood Neutrophils and Mononuclear Cells of mAD and AD Patients As Well As Healthy Controls

**Figure 3:**
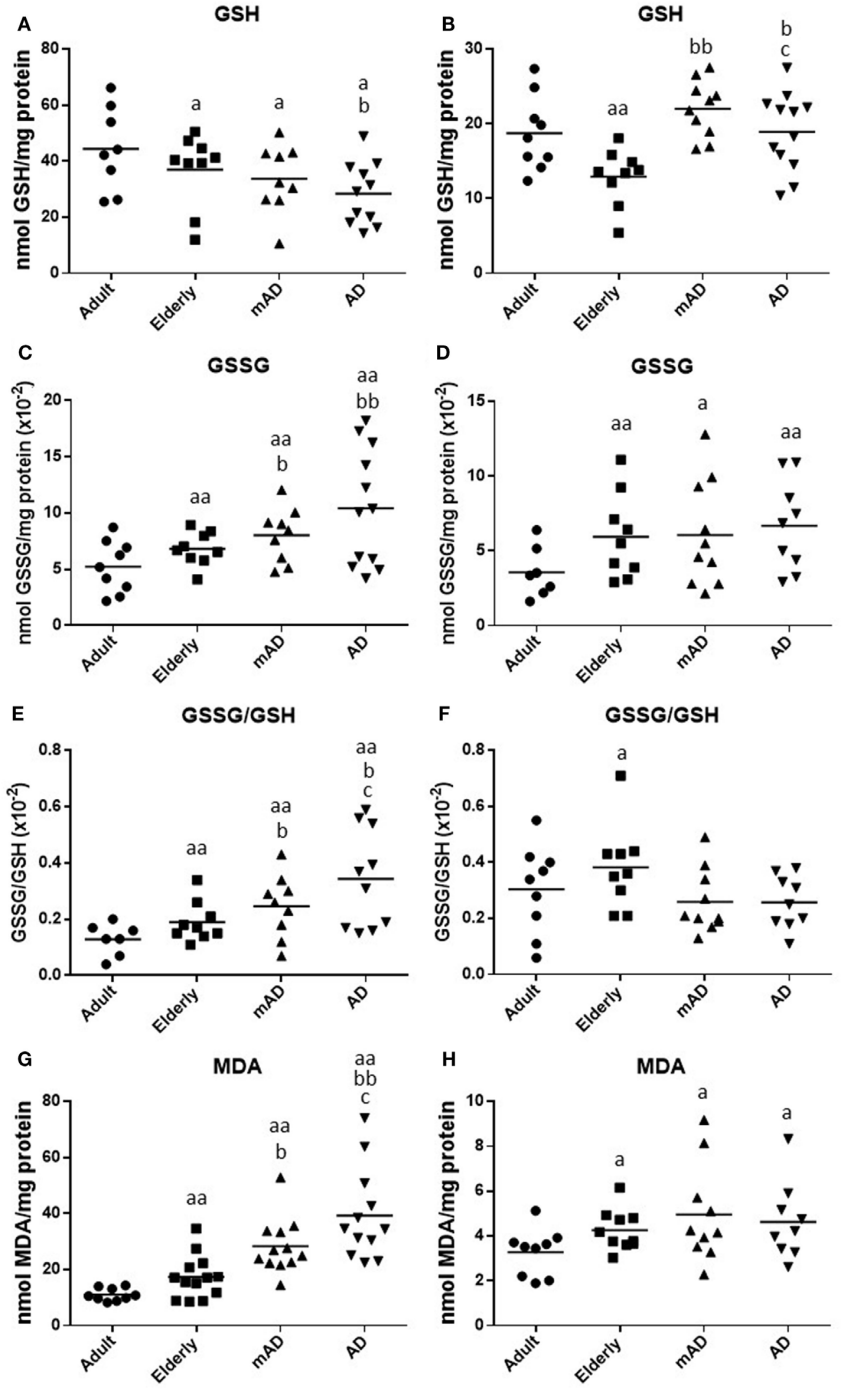
Oxidative stress parameters and lipid peroxidation in isolated peripheral blood neutrophils and mononuclear cells of mild Alzheimer’s disease (mAD) and Alzheimer’s disease (AD) patients, as well as of adult and elderly healthy subjects. Intracellular reduced glutathione (GSH) contents (nmol/mg protein) in neutrophils **(A)** and lymphocytes **(B)**; intracellular oxidized glutathione (GSSG) contents (nmol/mg protein) in neutrophils **(C)** and lymphocytes **(D)**; GSSG/GSH ratios in neutrophils **(E)** and lymphocytes **(F)**; and intracellular malondialdehyde (MDA) contents (nmol/mg protein) in neutrophils **(G)** and lymphocytes **(H)**. Data are shown as the mean (horizontal bar) of 9–12 values corresponding to the number of subjects analyzed in each group (9 adult, 10 elderly, 11 mAD, and 12 AD). Each value is the mean of duplicate assays. a: *P* < 0.05 and aa: *P* < 0.01 with respect to the value in adult subjects; b: *P* < 0.05 and bb: *P* < 0.01 with respect to the value in elderly subjects; and c: *P* < 0.05 with respect to the value in mAD patients.

A good maintenance of the redox state is vital for the proper functioning of immune cells, and thus the oxidative stress, which is the basis of aging and age-related diseases, is detrimental for leukocyte functions (28). Hence, the decline in immune response observed in mAD and AD patients could be mediated by an increase in oxidative stress and the consequent oxidative damage. To test this possibility, the intracellular content of GSH and GSSG, the GSSG/GSH ratio (one of the best markers of oxidative stress), as well as the MDA levels (one of the major lipid peroxidation products) were assessed in isolated human blood neutrophils and mononuclear cells obtained from mAD and AD patients, as well as from adult and elderly healthy subjects. The results are shown in Figure 3. In general, in both leukocyte populations a higher oxidative stress and damage have been shown in elderly subjects and AD patients than in adult controls. However, a different pattern of impaired redox state has been observed between the early and advanced stages of AD, the neutrophils showing a greater oxidative stress and damage than the lymphocytes.

Regarding aging, elderly healthy controls showed in their neutrophils and lymphocytes significantly lower GSH contents (*P* < 0.05 and *P* < 0.01, respectively; Figures 3A,B), as well as higher GSSG amounts (*P* < 0.01; Figures 3C,D), higher GSSG/GSH ratios (*P* < 0.01 and *P* < 0.05, respectively; Figures 3E,F), and MDA levels (*P* < 0.01 and *P* < 0.05, respectively; Figures 3G,H) in relation to adult subjects.

Regarding the AD pathology, AD patients had in their neutrophils a marked increase in both GSSG and MDA contents (*P* < 0.01; Figures 3C,G), as well as in GSSG/GSH ratios (*P* < 0.05; Figure 3E) in comparison to elderly subjects, accompanied also by a marked decrease in the levels of GSH (*P* < 0.05; Figure 3A). However, in the case of lymphocytes, AD patients showed higher GSH contents (*P* < 0.05; Figure 3B) than elderly controls, whereas no differences were observed in the other parameters analyzed. Likewise, similar results were observed in mAD patients, who showed higher GSSG contents, GSSG/GSH ratios, and MDA levels (*P* < 0.05; Figures 3C,E,G) in neutrophils as well as higher GSH contents (*P* < 0.01; Figure 3B) in lymphocytes, than the corresponding values in elderly controls. In relation to the different stages of AD, it is important to note that AD patients had in their neutrophils markedly increased GSSG/GSH ratios and MDA levels (*P* < 0.05; Figures 3E,G) as well as decreased GSH contents in their lymphocytes (*P* < 0.05; Figure 3B) in relation to mAD patients.

### Oxidative Stress and Damage Parameters in Human Peripheral Blood Cells of mAD and AD Patients as well as of Healthy Controls

**Figure 4:**
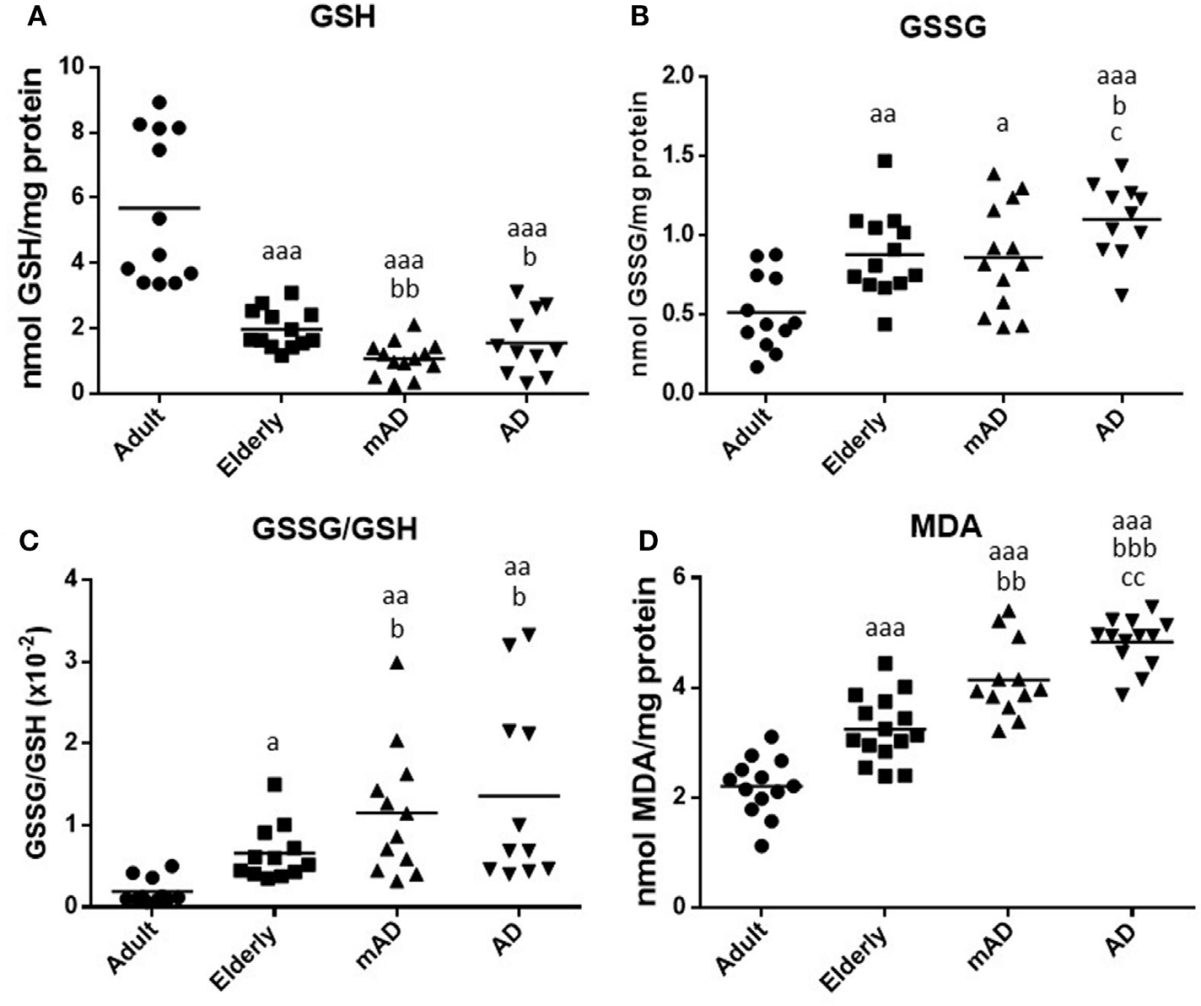
Oxidative stress parameters and lipid peroxidation in whole blood cells (containing both total red blood cells and leukocyte populations) of mild Alzheimer’s disease (mAD) and Alzheimer’s disease (AD) patients, as well as of adult and elderly healthy subjects. Intracellular reduced glutathione (GSH) contents (nmol/mg protein) **(A)**; intracellular oxidized glutathione (GSSG) contents (nmol/mg protein) **(B)**; GSSG/GSH ratios **(C)**; and intracellular malondialdehyde (MDA) contents (nmol/mg protein) **(D)**. Data are shown as the mean (horizontal bar) of 11–15 values corresponding to the number of subjects analyzed in each group (13 adult, 15 elderly, 13 mAD, and 13 AD). Each value is the mean of duplicate assays. a: *P* < 0.05, aa: *P* < 0.01, and aaa: *P* < 0.001 with respect to the value in adult subjects; b: *P* < 0.05, bb: *P* < 0.01, and bbb: *P* < 0.001 with respect to the value in elderly subjects; and c: *P* < 0.05 and cc: *P* < 0.01 with respect to the value in mAD patients.

It is known that there is a significant difference between the use of WB and purified and isolated PMNs and mononuclear leukocytes (62). Moreover, various peripheral blood cell types and their proportions can affect the immune response, as well as the inflammatory and oxidative stress conditions (54, 62). For this reason, the same parameters of oxidative stress and damage analyzed in isolated neutrophils and lymphocytes from mAD and AD patients, and adult and elderly healthy subjects, were also evaluated using samples of WB cells, containing both total RBC and leukocyte populations. This kind of sample better reproduces the *in vivo* conditions. The results are shown in Figure 4. There were higher values of oxidative stress and damage in blood cells of elderly subjects than in those of adults. Thus, GSH values were lower (*P* < 0.001; Figure 4A) and GSSG/GSH ratios higher (*P* < 0.05; Figure 4C), as well as both GSSG (*P* < 0.01; Figure 4B and MDA (*P* < 0.001; Figure 4D) levels, than those in adult subjects.

Regarding AD pathology, AD patients exhibited, in their blood cells, significantly lower GSH contents (*P* < 0.05; Figure 4A), higher GSSG and MDA contents (*P* < 0.05 and *P* < 0.001, respectively; Figures 4B,D), and GSSG/GSH ratios (*P* < 0.05; Figure 4C) than elderly subjects. Similar results were also observed in mAD patients in relation to elderly subjects, which also showed lower GSH levels (*P* < 0.01; Figure 4A) and higher GSSG/GSH ratios (*P* < 0.05; Figure 4C) and MDA contents (*P* < 0.01; Figure 4D) than elderly controls. However, no differences were observed in GSSG contents between mAD and elderly subjects. Finally, regarding the differences between early and advanced stages of AD, it is important to note that AD patients showed higher levels of GSSG and MDA (*P* < 0.05 and *P* < 0.01, respectively; Figures 4B,D) than mAD patients.

### Basal Cytokine Production in Blood Leukocytes of mAD and AD Patients as well as of Healthy Controls

Since an age-related increase in release of pro-inflammatory cytokines in resting cells leads to a sterile inflammation (30), and this alteration of the inflammatory status in aging (“inflamm-aging”) could lead to a chronic situation causing neuronal impairment and loss associated with AD (63), we also assessed extracellular release of IL-6, TNF-α, and IL-10 secreted *ex vivo* by blood cells cultured (4 h) under resting conditions, from mAD and AD patients, as well as from adult and elderly healthy controls.

As shown in Table 3, under basal conditions, in elderly subjects the values of IL-6 and TNF-α released were higher (*P* < 0.01 and *P* < 0.05, respectively) than in adults. Regarding AD pathology, a similar cytokine profile was also observed in AD patients in relation to both adults and elderly subjects. Thus, AD patients showed higher levels of IL-6 and TNF-α (*P* < 0.05) than elderly controls, whereas mAD patients only showed statistically significant higher values (*P* < 0.05) in the TNF-α levels. Basal IL-10 release was not detectable by the multiplex, so the IL-10/TNF-α ratio could not be calculated. Interestingly, regarding differences between early and advanced stages of AD, AD patients showed higher basal IL-6 release (*P* < 0.05) than the individuals with mAD.

**Table 3:** Levels of interleukin (IL)-6 and tumor necrosis factor (TNF)-α (pg/mL) released by peripheral whole blood cells of adult and elderly healthy subjects, as well as of mild Alzheimer’s disease (mAD) and severe Alzheimer’s disease (AD) patients, after 4 h of culture in basal conditions.

Basal cytokines	Adult	Elderly	mAD	AD
IL-6 (pg/mL)	8.89 ± 1.93	16.23 ± 4.72^a^	16.21 ± 3.84^a^	21.60 ± 5.17^b^^,^^c^^,^^d^
TNF-α (pg/mL)	24.76 ± 6.14	40.30 ± 14.89^e^	90.76 ± 26^a^^,^^c^	105.8 ± 22.5^a^^,^^c^

*Data are expressed as mean ± SD of the values corresponding to 10 adult, 10 elderly, 8 mAD, and 8 AD subjects. Each value is the mean of duplicate assays*.

*^a^P < 0.01 with respect to the value in adult subjects*.

*^b^P < 0.001 with respect to the value in adult subjects*.

*^c^P < 0.05 with respect to the value in elderly subjects*.

*^d^P < 0.05 with respect to the value in mAD patients*.

*^e^P < 0.05 with respect to the value in adult subjects*.

## Discussion

To our knowledge, this is the first study that analyzed the changes in several parameters of function and oxidative-inflammatory stress state in different types of peripheral blood immune cells, such as neutrophils and mononuclear cells, from patients with mild and severe AD, compared with a healthy age-matched control group of subjects without cognitive impairment. Additionally, all these parameters were also studied in healthy adult subjects to evaluate the differences due to the aging process. Our results revealed an impairment of the immune functions of both neutrophils and mononuclear cells at different stages of AD. Several alterations were only observed in severe AD patients and some only in mAD patients. Furthermore, our results also demonstrated that immune blood cells from mAD and AD patients, and especially neutrophils, showed an increased oxidative stress and oxidative damage in relation to elderly subjects. This altered redox balance could be mediated by the higher production of pro-inflammatory cytokines, which has been also observed in AD patients.

The immune system has a profound implication in the pathology of AD, not only at the central level but also at the peripheral level (8–10, 17, 19). Although several studies have reported alteration of both innate and acquired immunity at different stages of AD (12), there are many contradictory results (11–15, 17). Moreover, most of these studies in human and animal models have focused on the alteration of the adaptive immune response. However, little is known about the changes in innate immunity, especially those carried out by phagocytes (e.g., neutrophils). Our findings revealed that severe AD patients show lower neutrophil and lymphocyte chemotaxis, PHA- and LPS-lymphoproliferation, as well as higher basal lymphoproliferation in comparison to elderly healthy subjects. These AD-related changes are similar to those with aging (28, 30), which was also observed in the present study, although more exacerbated. This shows the presence of a lesser competent immune system in severe AD patients than in elderly subjects, which could contribute to the high mortality of these individuals. Our results agree with some of those observed in humans and experimental animals with AD (11, 16, 17, 26, 33, 52). Thus, leukocytes of 3xTg-AD mice presented an impairment of chemotaxis capacity (16, 26, 52). In relation to lymphoproliferative response in stimulated conditions some studies also report a decrease in this function in cells obtained from patients with severe AD (11, 17, 33, 64).

Surprisingly, mAD patients, but not severe AD patients, showed higher values in neutrophil and lymphocyte adherence than elderly subjects. This increase in adherence is a characteristic of aging (24, 28). Although at this moment we cannot explain the possible regulation mechanisms that severe AD patients use to present similar values to age-matched controls, this parameter could be an early marker of the appearance of AD. In mAD patients, a lower proliferative response of lymphocytes to the mitogen PHA than in elderly subjects also occurred. Thus, this function of lymphoproliferative response to mitogens, which typically decreases with aging (24, 28, 30) appeared even lower in cells of mAD and AD.

Alzheimer’s disease patients in comparison to mAD showed lower lymphocyte chemotaxis and antitumoral NK activity. In this regard, although limited in number, several studies in human and 3xTg-AD mice revealed that these immune function parameters were altered in AD (16, 26, 32, 52). Thus, NK cell activity, which has been proposed as one biomarker of immune alterations in the progression of AD (32) and which was decreased in cells of 3xTg-AD mice in advanced stages of AD (16, 26), was lower in severe AD than in mAD patients. In the case of chemotaxis of lymphocytes, a function that decreases with aging (28, 30), our results reflect a clear deterioration of this activity in AD patients, even higher than shown by cells of elderly subjects and of mAD. Thus, this parameter could be considered as a possible marker of the progression of the disease.

Interestingly, it should be noted that although the decline of the immune functions showed with aging were also observed in mAD and AD patients, several of these immunological alterations were greatly exacerbated in individuals with severe AD in comparison to healthy elderly subjects of the same chronological age. This suggests that AD patients suffer an accelerated immunosenescence. Thus, since these immune function parameters are good markers of health and predictors of longevity (28, 30), these could be used as peripheral biomarkers of the progression of AD.

A chronic oxidative-inflammatory stress, a condition in which oxidant and pro-inflammatory compounds overwhelm antioxidant and anti-inflammatory defenses, is associated with aging and several age-related pathologies (26, 28). Indeed, several studies in human and animal models, including some by our group, have demonstrated that increased peripheral oxidative stress markers are associated with aging and, more specifically, with AD (16, 26, 30, 47, 52, 54). In AD, oxidative stress has been recognized as an essential contributor to the pathogenesis and progression of the disease (38, 40, 52, 65). As occurs in the brain (7, 40), an enhanced oxidative stress during AD is also present in peripheral blood cells (27, 41–43). Most studies have focused on mononuclear cells, mainly in lymphocytes, which reflect the pathological oxidative stress conditions observed in the brains of AD patients. These studies found an elevated production of oxidative compounds, lipid oxidation, DNA damage, mitochondrial susceptibility, basal apoptosis as well as altered levels of antioxidant enzymes in lymphocytes of AD patients, as well as even in those of MCI subjects, in comparison with these cells of healthy subjects (27, 66–68). These results suggest that some of these parameters could be prodromal markers of AD. However, little is known about the changes in the redox balance of neutrophils during the progression of AD. Therefore, since phagocytes may be the immune cells that contribute most to the oxidative stress and damage associated with immunosenescence (28, 54), we also analyzed different redox state and oxidative damage parameters in both isolated peripheral blood neutrophils and mononuclear cells, in order to elucidate possible differences between them. Interestingly, our results showed that neutrophils suffer a marked increased oxidative stress and damage during the progression of AD. In particular, the neutrophils of both mAD and AD patients had lower antioxidant GSH contents and higher oxidant markers, such as GSSG and MDA contents, as well as GSSG/GSH ratios, than elderly subjects. Nevertheless, this redox state alteration was not observed in mononuclear cells, which paradoxically showed higher GSH levels in individuals with mild and severe AD than those observed in age-matched controls. In this context, it has been suggested that in early states of the disease, individuals developing AD can suffer a situation of “reductive stress” as a result of the activation of a compensatory response to cope with a high exposure of oxidant compounds (40). Therefore, targeting reductive stress may be a strategy to delay and prevent the onset and progression of AD (69). Thus, it is plausible that an overexpression and high levels of GSH could be induced by an increased oxidant production in lymphocytes of mAD patients. Nevertheless, these levels were lower in severe AD patients in comparison to mAD subjects. The glutathione cycle is one of the main intracellular mechanisms to preserve a competent intracellular redox state (70). The role of this has been extensively studied in AD, in both humans and animal models, where a depletion in GSH contents and an imbalance in GSSG/GSH ratios in favor of GSSG have been noted at central (39) and peripheral levels, such as in plasma, peritoneal leukocytes, and blood cells (47, 52, 71). Moreover, it has been described that the altered levels of these circulating compounds are also directly related to the severity of cognitive impairment (47, 67). The imbalance in GSSG/GSH ratios could be the result of a defective respiratory chain caused by the activation of Aβ peptide on its complexes, which leads to overproduction of oxidant compounds, which has also been observed in peripheral leukocytes (72). In addition, an adequate immune response will require optimal levels of GSH (70). Therefore, the lower GSH observed in neutrophils of AD could contribute to the impairment of the functions of these cells.

Since the brain is very susceptible to oxidative damage (36), lipid peroxidation is one of the most promising procedures in AD diagnosis (47). Thus, higher MDA content has been reported in the brain and the peripheral levels (e.g., plasma/serum, erythrocytes, and peripheral leukocytes) of not only AD patients but also MCI subjects (42, 46, 47). Some authors suggested that lipid peroxidation could be one of the main factors responsible for cognitive deterioration and that there was a negative correlation between MDA and MMSE scores (43). Others reported that the differences depended on the stage of AD (42, 43, 47). However, although no difference between moderate and advanced AD was detected (46), our results showed that MDA levels in neutrophils were higher in patients at the advanced stage of the disease than in elderly controls and even in mAD. Thus, this parameter could be a useful marker of early stage of AD as well as progression of the disease.

Given the different pattern observed in the oxidative stress and damage parameters between neutrophils and mononuclear cells in the progression of AD, we have also analyzed these parameters in WB cells. This kind of sample better reproduces the *in vivo* conditions of immune response. Interestingly, a similarly altered redox state and oxidative damage pattern were observed in WB cells to that in neutrophils. Thus, mAD and AD patients, in general, showed lower GSH and higher GSSG contents and GSSG/GSH ratios, together with a marked increase in MDA levels, than cells from elderly subjects. Moreover, it is important to highlight that AD patients had a higher GSSG/GSH ratios, as well as GSSG and MDA contents, in their neutrophils and WB cells than mAD patients. The results suggest that the estimation of these parameters in WB cells would be a useful biomarker in the assessment of AD progression. Moreover, WB samples are clinically more feasible, reproducible, cost effective, easy to implement and apply, compared to purified and isolated neutrophils and mononuclear blood leukocytes.

In addition, since oxidation and inflammation processes are able to induce and exacerbate one another (36), the higher oxidative stress and damage observed in WB cells from mAD and AD patients could also be triggered by an increase in basal inflammation. Given that previous works supported the importance of cytokines in mediating the activity of peripheral immune cells in AD (73, 74), we also assessed the release of IL-6, TNF-α, and IL-10 *ex vivo* by blood cells cultured under resting conditions. Our results showed that mAD and AD patients had a higher basal TNF-α than elderly subjects, whereas basal IL-6 levels only were significantly higher in severe AD. Basal IL-10 release was not detectable. Even though cytokine levels are hard to detect at basal conditions, due to their short half-life, several studies also investigated these inflammatory markers in CSF, serum and plasma at different stages of the AD patients (73–76). Although the results are controversial, a strong upregulation of pro-inflammatory cytokines has been observed in these patients, but also in MCI (73, 74, 76, 77). Furthermore, these patients showed higher plasma levels of IL-6 than those at an early stage of disease and healthy controls (77). This agrees with our results, in which severe AD patients also showed higher IL-6 release than mAD. Interestingly, given that increased basal IL-6 levels in plasma have been shown to be a risk for decline in cognitive functions (73), as well as being described as one the most powerful predictors of morbidity and mortality in the elderly (30), these results suggest the possibility of using peripheral IL-6 production as a helpful tool to characterize immune dysfunction during AD progression.

The chronic oxidative-inflammatory stress situation observed in blood cells of AD patients could be involved in the impairment of their functions. Thus, the higher oxidative stress in severe AD patients could be the underlying mechanism for the decrease in PHA- and LPS-induced lymphoproliferation, as well as for the increase in the basal proliferation. This basal proliferation, which has scarcely been studied, was also increased with aging (30), as well as in the leukocytes of 3xTg-AD mice (52). The higher levels of basal proliferation in AD lymphocytes show an overactivation of these cells. This could be due to the increase age-related accumulation of damage-associated molecular patterns, which, through the production of pro-inflammatory cytokines, may be responsible for the activation of the immune system and the establishment of “sterile inflammation” (78). In addition, after a mitogenic LPS-stimulus, immune blood cells from AD patients also produced a higher release of IL-6 and TNF-α, as well as lower IL-10 levels and, consequently, a lower IL-10/TNF-α ratio, compared to age-matched controls. This reaffirms the existence of an altered immune response in advanced stages of AD. Although data on peripheral cytokines in AD patients are controversial (73, 74), several authors also found an upregulation of these cytokines in LPS-stimulated WB in AD patients in relation to elderly controls (75), as also occurred in samples of brain, serum, and other cell cultures (79). A possible explanation would be that βA peptide, which can activate the overproduction of pro-inflammatory cytokines (e.g., TNF-α, IL-1β, etc.) in peripheral blood monocytes (80, 81), could lead to an abnormal inflammatory response. This could be attributed either to an adaptive control on innate immunity and/or a compensatory mechanism for the lack of effectiveness of adaptive immunity (81). Our results also demonstrated an altered balance in the IL-10/TNF-α ratios, which showed similar values in mAD patients and elderly subjects, whereas the values were lower in AD patients. Since this IL-10/TNF-α ratio has been proposed as an indicator of successful aging and longevity (30), its determination also may be a helpful parameter for evaluating the progression of AD. Since the decline in PHA-lymphoproliferation and the high release of IL-6 after LPS stimulation not only occur in severe AD patients but also begin to decline in mAD patients, this suggests that both parameters could be used as possible early peripheral biomarkers of AD.

In conclusion, the present study shows the impairment of several immune functions of human peripheral blood neutrophils and mononuclear cells at different stages of AD. However, a different pattern of altered immune response was observed between mild and severe AD patients. Thus, several alterations were only observed in severe AD patients (e.g., chemotaxis, basal, and LPS lymphoproliferation) and others (e.g., adherence) only in individuals with mAD. Other alterations detected in the mild stage of the disease increased in the late stage (e.g., PHA lymphoproliferation and IL-6 release). As occurs in aging, this impairment of immune cell functions could have as their basis an oxidative-inflammatory stress situation. Thus, our findings also demonstrated that several peripheral oxidative stress and damage markers were increased in immune blood cells from mAD and AD patients, especially in neutrophils (e.g., low GSH levels, high GSSG/GSH ratios, and GSSG and MDA contents), in relation to elderly subjects. However, this increased oxidative stress and damage were higher in severe AD patients than in mAD and could be mediated by the higher production of peripheral pro-inflammatory cytokines in these patients. Furthermore, the fact that neutrophils showed higher oxidative stress and damage than mononuclear leukocytes supports the idea that phagocytes are the immune cells that most contribute to the peripheral chronic oxidative stress and damage associated with AD, especially in the advance stages of the disease. Therefore, our results support the idea that severe AD patients show an accelerated immunosenescence due to the parameters of function and redox state studied, these being greatly deteriorated in these patients in comparison to elderly subjects of the same chronological age. Therefore, since WB cells are very easy to obtain and reproduce the results observed in neutrophils, the assessment of oxidative stress and damage parameters, as well as peripheral cytokine release, together with the analysis of several functions in isolated neutrophils and mononuclear cells, would be useful markers of AD progression. Nevertheless, additional studies are needed to identify function and oxidative-inflammatory stress alterations in peripheral blood phagocytes and lymphocytes, especially in subjects with mild cognitive deterioration in order to identify potential prodromal and preclinical biomarkers of AD.

## Ethics Statement

All procedures were carried out according to the Declaration of Helsinki, and approval was obtained from the corresponding Research Ethic Committees.

## Author Contributions

MF—formulated the original problem, designed the work, provided direction and guidance, wrote the manuscript and critically reviewed the final version of the manuscript. CV—provided input for experimental design, carried out experiments with patient samples, analyzed data results, and wrote the manuscript; IT—provided input for experimental design and carried out experiments with patient samples; AG—carried out experiments with patient samples; EC and JM provided the samples for the experimental study; and JM made the clinical diagnosis of Alzheimer’s disease patients and control’s selection.

## Conflict of Interest Statement

The authors declare that the research was conducted in the absence of any commercial or financial relationships that could be construed as a potential conflict of interest. The reviewer IT and handling Editor declared their shared affiliation.
